# Wnt/β-Catenin Signaling Pathway in Pediatric Tumors: Implications for Diagnosis and Treatment

**DOI:** 10.3390/children11060700

**Published:** 2024-06-07

**Authors:** Sahar Choudhary, Mithalesh Kumar Singh, Seema Kashyap, Rachna Seth, Lata Singh

**Affiliations:** 1 Department of Pediatrics, All India Institute of Medical Sciences, New Delhi 110029, India; sahar.aiims31@gmail.com (S.C.); drrachnaseth1967@gmail.com (R.S.); 2 Department of Ophthalmology, UT Southwestern Medical Center, Dallas, TX 75390, USA; 3 Department of Ocular Pathology, All India Institute of Medical Sciences, New Delhi 110029, India; dr_skashyap@hotmail.com

**Keywords:** Wnt signaling pathway, neuroblastoma, rhabdomyosarcoma, retinoblastoma, Wilms tumor, targeted therapies

## Abstract

The evolutionarily conserved Wnt signaling has a significant and diverse role in maintaining cell homeostasis and tissue maintenance. It is necessary in the regulation of crucial biological functions such as embryonal development, proliferation, differentiation, cell fate, and stem cell pluripotency. The deregulation of Wnt/β-catenin signaling often leads to various diseases, including cancer and non-cancer diseases. The role of Wnt/β-catenin signaling in adult tumors has been extensively studied in literature. Although the Wnt signaling pathway has been well explored and recognized to play a role in the initiation and progression of cancer, there is still a lack of understanding on how it affects pediatric tumors. This review discusses the recent developments of this signaling pathway in pediatric tumors. We also focus on understanding how different types of variations in Wnt signaling pathway contribute to cancer development and provide an insight of tissue specific mutations that lead to clinical progression of these tumors.

## 1. Introduction

Wnt (Wingless-related integration site) signaling is an evolutionarily conserved pathway across all genera of animal kingdom [1]. It was first identified about 40 years ago and is one of the oldest development signaling pathways. Roel Nusse and Harold Varmus discovered Wnt signaling in 1982 after infecting mice with the mouse mammary tumor virus in order to mutate mouse genes that could cause breast tumors [2]. They identified a new mouse proto-oncogene that they named int1 (integration 1). It was discovered that the gene products of the mouse proto-oncogene Int1 (now known as Wnt1) and Drosophila melanogaster wingless (wg) are orthologous during the initial study for Wnt signal transduction in the 1980s and 1990s. “Wnt1” is a combination of the terms “wingless” and “Int1” [3].

A single event in the Wnt axis interlinks and iterates a series of molecular signals in the cell. The amplification of these signals will control cell fate by inducting cell survival or apoptotic signal [4]. Additionally, the Wnt pathway has a myriad of functions to play from cell regeneration to shaping cells and maintaining tissue structure by conferring cell polarity and cell asymmetry, resulting in cell diversification [5,6,7]. Wnt signaling participates in all kinds of cellular processes, including proliferation, differentiation, migration, invasion, and cell and tissue homeostasis [8]. Owing to its vigilant role in all cellular processes, deregulations in this pathway contribute to development and progression of many diseases. There is growing evidence explaining its role in developmental disorders as well as cancer [9,10,11,12,13].

**Figure 1:**
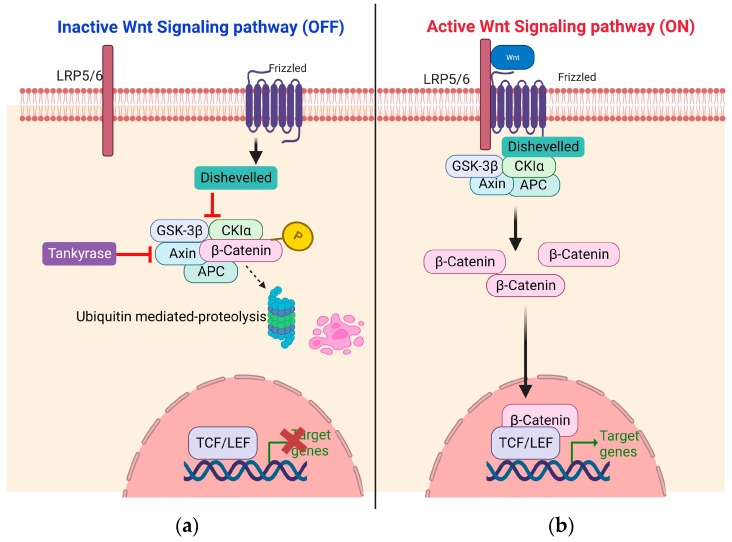
Canonical Wnt signaling pathway. (**a**) Inactive Wnt signaling pathway (OFF): When there are no Wnt ligands bound to the frizzled protein (FZD), the multiprotein destruction complex (adenomatous polyposis coli (APC), the glycogen synthase kinase 3β (GSK3β), axin, and the casein kinase 1α (CK1α)) that reside in the cytoplasm bind to and phosphorylate β-catenin, which leads to proteasomal degradation. This prevents the translocation of β-catenin in nucleus, where T-cell factor/lymphoid enhancer factor (TCF/LEF) alone halts the transcription of target genes. (**b**) Active Wnt Signaling pathway (ON): When there are Wnt ligands bound to the frizzled protein and LRP5/6 co-receptors, the destruction complex is inhibited and that allows for the accumulation and stabilization of β-catenin in the cytoplasm and subsequent translocation into the nucleus. The β-catenin-TCF/LEF complex drives the expression of target genes.

Wnt proteins act as intercellular signals that regulate the proliferation of cells. Wnt proteins have discreetly unique properties when compared to other growth signals. They have a short range of action and act locally to mediate signals among neighboring cells [14]. The Wnt pathway occurs via two mechanisms, the non-β-catenin dependent known as non-canonical and β-catenin dependent known as canonical Wnt signaling pathway. There is ample evidence explaining the role of canonical Wnt signaling pathway in multiple cancers (Figure 1).

**Figure 2:**
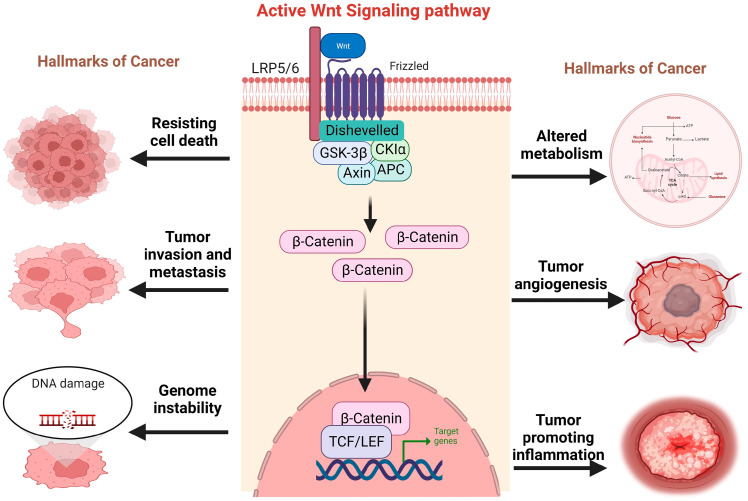
This figure demonstrates the role of the Wnt signaling pathway associated with numerous cancer hallmarks that contribute to cancer development and other signaling networks.

A continuous degradation of β-catenin occurs via the formation of a “destruction complex” comprising different proteins including Axin, which is a scaffolding protein, the adenomatous polyposis coli (APC) protein, casein kinase 1 (CK1), and the Ser/Thr kinase glycogen synthase 3 (GSK3). This destruction complex acts as the main control center of canonical Wnt pathway. In the absence of any external Wnt signaling, GSK3 phosphorylates β-catenin, and it becomes fated for proteasomal degradation [15]. Contrarily, binding of the Wnt ligands to a receptor complex of Frizzled proteins (FZD) and the coreceptor low-density lipoprotein receptor-related protein 5/6 (LRP5/6) inhibits β-catenin destruction by activating disheveled (DVL), which phosphorylates and deactivates GSK3. Hence, β-catenin is not phosphorylated, leading to its cytoplasmic accumulation and nuclear translocation. β-catenin binds to transcriptional factor complex T cell factor/lymphoid enhancing factor 1 (TCF/LEF1) by replacing the corepressor TLE to activate the transcription of various target genes such as c-Myc, cyclin D1 and CDKN1A [16]. Wnt/β-catenin signaling affects a number of pathways and cancer hallmarks that control the development and progression of cancer (Figure 2).

## 2. Pediatric Tumors

Pediatric tumor etiology and pathology are less complex as children undergo less mutational hits in their genetic material as compared to adults. The age range encompasses infancy, early childhood, and pre-adolescence. The age range of 0–14-year is used to define children in some countries and global health organizations, and adolescent age is considered to be any person between the ages of 10 and 19 years, according to the World Health Organization [17]. While many adult tumors originate due to sedentary lifestyle and other risk factors [18], recent advances in cancer genomics have prompted the WHO to redefine the classification of pediatric tumors, which takes into consideration the transcription profiles, mechanism of tumorigenesis, and clinical outcomes. This classification aims to redesign the treatment regimen for children with cancer to improve survival and quality of life in the long run [19].

The Wnt signaling pathway directs growth and cell patterning during early embryogenesis. Mutations in Wnt-pathway-associated genes cause aberrant cell proliferation and chromosome instability that lead to specific developmental defects and carcinogenesis. Increased β-catenin levels may also promote neoplastic conversion by triggering cyclin D1 gene expression, uncontrolled progression into the cell cycle [20], and has been implicated to drive early events in tumorigenesis. Most pediatric tumors are known to occur in developing tissues while they are undergoing substantial expansion, growth, and maturation. A genomic analysis revealed multiple alterations in the Wnt-pathway-associated genes that generate oncogenic signals in pediatric tumors [21].

As per an extensive review of the literature, the influence of gender on the Wnt/β-catenin signaling pathway might play an important role in pediatric tumors. There are studies that have shown the effect of differential expression of Wnt/β-catenin genes on age and gender in human bone marrow stromal cells (hMSCs). One such study indicated that Wnt2 and Wnt13 showed a trend of higher expression in hMSCs from young group whereas expression of Wnt7B, 13, and 14 were inversely correlated with age. Gender-specific differences indicated that Wnt16 expressed significantly higher in men whereas Wnt11 showed higher expression in hMSCs from women. However, there are no studies that have demonstrated the effect of differential expression of Wnt genes on age and gender in pediatric population [22]. Hence, the role of the ‘Wnt/β-catenin signaling pathway’ genes in the development of children and how they might correlate to the development and prognosis of various tumors is an intriguing topic to explore.

## 3. Implications of Wnt/b-Catenin Signaling Pathway in Pediatric Tumors

Recent research has shown that the Wnt pathway controls the developmental process, tissue homeostasis, cell proliferation, survival, migration, as well as maintenance of the stem cells. Activation of this pathway is known to initiate organ formation and expansion. Some recent studies have identified mutations in this pathway that drive tumorigenesis and massive cell proliferation in various solid tumors. Alterations in the genetic landscape during germinal differentiation are the major cause of metastatic pediatric tumors. Most pediatric tumors are aggressive in nature and do not respond to local therapy. Systemic therapy is the choice of treatment for tumors with such substantial side effects. Treatment intensification has not improved the prognosis of patients, and that brings attention to the urgent need for novel approaches for the prevention and treatment of relapsed and refractory disease.

**Figure 3:**
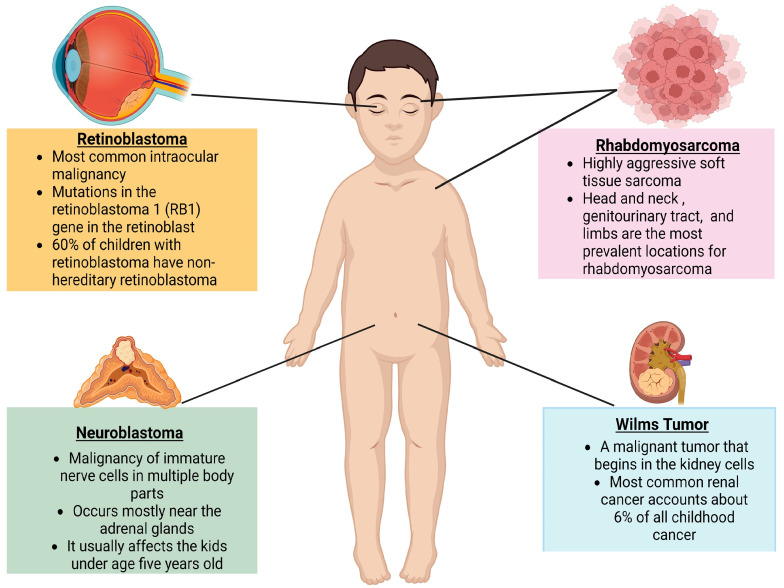
A schematic depicting the different types of pediatric tumors—Retinoblastoma (eye), rhabdomyosarcoma (soft tissue sarcoma), neuroblastoma (sympathetic nervous system) and Wilms tumor (kidneys).

It has been observed that the Wnt pathway is overactivated in relapse patients and the inhibition of Wnt signaling restores chemosensitivity in resistant disease in certain pediatric tumors. A crucial step in the transformation of normal cells is the stabilization and overproduction of β-catenin protein in multiple forms of cancer. This may be a result of active mutations in CTNNB1 gene [23]. As a result, an inhibitor that specifically targets a protein–protein interaction within the system may disrupt aberrant Wnt signaling in tumor cells while protecting Wnt signaling in healthy cells. This prevents tumor growth while maintaining healthy tissue development. Currently, small molecules that control various protein–protein interactions have been synthesized and characterized as regulators, inhibitors, and activators of Wnt signaling in various pediatric tumors. Therefore, there is increasing evidence that establishes the role of Wnt pathway in the development of multiple pediatric tumors such as retinoblastoma, neuroblastoma, rhabdomyosarcoma, and Wilms tumor, all of which will be discussed in this review in context to the β-catenin-dependent (canonical) Wnt signaling pathway (Figure 3).

## 4. Wnt Signaling in Retinoblastoma

Retinoblastoma (Rb) is the most common pediatric intraocular malignancy with an incidence rate of about 1 in 18,000 cases [24]. Currently, the treatment modalities for RB include enucleation, along with radiotherapy, brachytherapy, external beam chemotherapy, and systemic chemotherapy [25]. Therapeutic interventions have improved the overall survival rate of Rb patients but chemoresistance may lead to complications and even death [26]. Thus, decoding the mechanism of tumor progression is vital for the development of newer diagnostic biomarkers.

Rb has been linked to genomic structural variations within Wnt signaling pathways and tumor-related mutations in Wnt signaling-associated genes [27]. Studies have revealed that expression of Wnt genes in the retina contributes to altered Wnt signaling pathway in Rb.

Wnt signaling controls the proliferation and differentiation of non-neoplastic stem/progenitor cells in the retina and other tissues. Lithium chloride (LiCl), a Wnt pathway activator, increased the number of stem-like cells in Y79 and Weri-Rb1 cell lines and elevated the expression of those stem cell marker genes. This suggests that the Wnt pathway might be a potential mechanism for the control of stem cell renewal and tumor formation in Rb [28]. A study reported on the tumor suppressive function of APC-2 gene in both Rb tumor samples and Y79 cells. Reduction in APC-2 levels due to hypermethylation leads to an increase in β-catenin expression [29]. The Wnt/β-catenin pathway activator restores the anticancer effects of MEG3 in Rb. Overexpression of MEG3 inhibits proliferation and increases apoptosis via inhibition of the Wnt/β-catenin pathway in Rb cell lines [30]. MEG3 then promotes the breakdown of β-catenin by GSK-3β, inactivating the Wnt pathway, thereby inhibiting the migration and metastasis of retinoblastoma cells [31]. Niclosamide inhibits cell proliferation by hindering the cell cycle at the G2/M phase and inducing apoptosis via the caspase-dependent pathway in Y79, RB116, and WERI-Rb-1 cell lines by downregulating the expression levels of p-LRP6, DVL2, and β-catenin. Wnt activator lithium reverses the inhibitory effects of niclosamide in Y79 cells, establishing Wnt/β-catenin as a molecular target of niclosamide in Rb cells [32]. Wnt/β-catenin signaling pathway was also found to be negatively modulated by long non-coding RNA (lncRNA) MT1JP in the development of Rb [33].

HMGA1 and HMGA2 proteins were found to be expressed in Rb and predicts poor prognosis [34]. Studies have found that HMGA2 as a direct target gene of miR-98 inhibits the development of Rb by mediating EMT and Wnt/β-catenin pathway [35]. Dysregulation of lncRNA LEF1-AS1, and ZEPM2-AS1 are involved in the progression of Rb through the regulation of the Wnt/β-catenin pathway [36,37]. An epigenetic regulator, lncRNA muscleblind-like protein 1 antisense RNA 1 (MBNL1-AS1) has anti-tumor effects in many cancers, including bladder cancer, papillary thyroid cancer, and Rb. Overexpression of MBNL1-AS1 significantly downregulates the Wnt/β-catenin signaling pathway. Hence, MBNL1-AS1 can be exploited for its protective role against Rb. A recent study found that disheveled-Axin domain containing 1 (DIXDC1) was dysregulated in Rb and suggests its oncogenic role in development of Rb by inhibiting Wnt pathway. This study suggests that DIXDC1 can be targeted as a novel therapeutic option against Rb. Another molecule CircTET1 was identified to prevent Rb progression by sponging miR-492/miR-494-3p and interrupting the Wnt/β-catenin pathway [38].

Contradictorily, the tumor suppressor role of the Wnt pathway has been implicated in a study by Tell S et al., who demonstrated that Wnt signaling activation significantly decreased the proliferation and viability of cells by inducing cell cycle arrest in Y79, RB355, and Weri-Rb1 cell lines [39]. β-catenin was significantly downregulated with the treatment of indomethacin in Y79 cells, which suggests the implication of the Wnt/β-catenin pathway in Rb [40].

However, it has been well implicated that Wnt/β-catenin activation is essential for Rb survival and proliferation. While Silva et al. and Gao et al. established that Wnt/β-catenin signaling pathway positively controls the proliferation of Rb cells, the function of this pathway still needed to be further validated in Rb. On review of the literature, it might be assumed that targeting Wnt/β-catenin signaling pathway has a promising future therapeutic role in Rb patients [32]; however, there remains a gap in recent literature regarding the role of /β-catenin signaling pathway in Rb.

## 5. Wnt Signaling in Neuroblastoma

Neuroblastoma is one of the most common malignancies in children with a considerably high mortality rate in advanced stages. Neuroblastoma arises in the neural crest cells of the sympathetic nervous system. It presents with different clinical symptoms and is usually diagnosed at a later stage when the tumor has metastasized. Although the overall five-year survival rate is 75%, but it is less than 45% in high-risk neuroblastoma that represent about 40% of patients [20,41,42]. Amplification of the MYCN gene is associated with poor prognosis and low survival rate.

MYC, along with other genes like ALK, are the transcriptional targets of Wnt/β-catenin signaling pathway. MYCN upregulates proliferation and apoptosis that target the Wnt/β-catenin signaling pathway, and hence acts as the major factor in the development of neuroblastoma. Wnt/β-catenin signaling pathway plays an important role in embryonal development. Its implications in organogenesis and cancers have been widely studied and explained in previous studies. There are studies that describe how the highly conserved Wnt signaling pathway regulates the development and differentiation of the neural crest cells during embryogenesis. When Wnt signaling pathway is inhibited, it induces apoptosis and hinders proliferation and oncogenesis in all types of neuroblastic cells [43]. BORIS is a DNA-binding protein that recruits certain transcription factors and induces tumorigenesis. Crosstalk between BORIS and the Wnt signaling pathway in manipulating oncogenic networks has been shown to be involved in maintenance of stemness, regulation of metastasis and cellular proliferation in MYCN amplified the IMR-32 neuroblastoma cell line [44]. β-catenin also plays an important role in the development of pre-migratory neural crest cells, maintenance, growth, and proliferation of neural stem cells. These factors drive tumorigenesis in neural precursor cells. In KEGG database, NFATC1, FBXW11, TP53, AXIN2, LRP5, CCND1, FZD9, DVL2, FOSL1, WNT7B, VANGL1, LEF1, and PPP3CB gene mutation have been identified in neuroblastoma and found to have functional impact in Wnt pathway components [45]. High FZD1 expression mediates chemoresistance, FZD6 marks highly tumorigenic stem-like cells, and FZD2-dependent proliferation of neuroblastoma cells through activation of the Wnt/β-catenin pathway [46]. In another study, 90 Wnt target genes were identified that showed the association of Wnt signaling with numerous transcription factors and other signaling pathways in neuroblastoma [47].

Dysregulation of miRNAs and epigenetic regulators has also been observed in neuroblastoma. miR-492 plays a wide role in enhancing tumorgenicity in many forms of cancer. Previous research has demonstrated that miR-492 polymorphisms can contribute to cancer susceptibility. miR-494 plays a role in cell adaptability to oxidative stress in neuroblastoma cells [48]. A study by Wang et al. showed that there was no significant correlation between miR-492 rs2289030 G>C and the risk of tumorigenesis in a cohort of 402 neuroblastoma patients [49].

Delving through the mechanism of action and limitations of the current treatment helps in laying down a path for bench and bedside formation of novel therapies. An adjuvant therapy with retinoids in combination with surgery and chemotherapy had proven to be useful in high-risk neuroblastoma patients [50]. Retinoic acid is known to modulate both canonical and non-canonical Wnt signaling pathways. The group of neuroblastoma patients administered retinoic acid had a better three-year event-free survival rate than the group of patients that received no additional medication [51]. However, retinoids were being used only as maintenance therapy and its mechanistic role has not been explored in neuroblastoma patients at yet. Current chemotherapy for neuroblastoma in children includes chemotherapeutic agents, which cause myelosuppression or subsequent infection. Monoclonal antibodies (mAbs) against GD2 antigen have been approved by the Food and Drug Administration (FDA) to treat solid pediatric tumors, especially neuroblastoma. Many inhibitors targeting ALK, MYCN, RAS/MAPK, p53/MDM2, and PI3K/Akt/mTOR have cleared phase I/II of clinical testing [52]. However, most immunotherapeutic therapies remain at the early phases of clinical trials. Therefore, it is important to explore Wnt/β-catenin signaling pathway to identify better molecular targets for development of advanced therapies that would improve outcome and prolong patient survival in high-risk NB patients.

## 6. Wnt Signaling in Rhabdomyosarcoma

Rhabdomyosarcoma (RMS) is one of the most prevalent, extremely aggressive soft tissue sarcomas in children and occurs with a partially preserved skeletal muscle differentiation [53]. It has a yearly incidence of 4.3 cases per million in people below the age of 20 years [54]. Currently, multimodal therapy can cure about 70% of children with non-metastatic disease [55]. However, there is no effective treatment for patients with relapsed embryonal RMS (ERMS), with less than 50% surviving the disease. There is currently a lack of information on how this disease affects developing countries including India. Alveolar RMS (ARMS) and ERMS are the two primary histological RMS subtypes that have been identified. These subgroups differ in disease prognosis and progression, as well as in their histological appearance and molecular features [56,57]. It is of high priority to identify molecular biomarkers that can diagnose and monitor the development of this disease and understand its pathogenesis.

Although the canonical Wnt/β-catenin signaling pathway plays a significant role in skeletal muscle development, it is unclear from the scant data how this pathway transforms these cells into RMS. This could be because RMS rarely exhibits nuclear β-catenin and does not exhibit mutations in important pathway components [58]. Dysregulation of Wnt signaling pathway is essential for myogenesis that contributes to the tumorigenesis of RMS and known to have a tumor suppressive effect in this neoplasia. According to Sing S and Eleanor Chen et al., induction of the canonical Wnt/β-catenin pathway induces differentiation of tumor-propagating cells and reduces cell growth and self-renewal in RMS [59]. A group conducted a proteomic analysis to identify certain differential proteins (DPs) as a diagnostic and prognostic biomarker for RMS. Interestingly, the DPs found in this study were mostly involved in the regulation of Wnt/β-catenin signaling, PI3K/AKT signaling, and FAT10 cancer signaling pathways, which modulate the process of cell differentiation and proliferation [60]. Another study reveals disrupted Wnt signaling genes enhance the growth of ERMS with extremely invasive characteristics in p53/c-fos double mutant mice [59]. In a similar way, activation of the classical Wnt/β-catenin pathway via inhibition of GSK3 prevents development and regeneration in ERMS [61]. There are few studies in the literature which implicate its direct role in invasion, angiogenesis, and metastasis in RMS [60].

Some studies suggest that non-canonical Wnt signaling is more significant in RMS than the canonical Wnt/β-catenin pathway, which appears to play a secondary function in RMS. Ragab et al. investigated the role of WNT5A, a major ligand of non-canonical WNT signaling in ERMS and ARMS, which antagonizes the canonical Wnt pathway [62]. When stably overexpressed in RMS cell lines, WNT5A decreased cell proliferation, migration, and self-renewal capacity. It also affects the expression of stem cell markers and modulates the levels of muscle differentiation markers by sphere assay and western blot analysis, respectively. Gene expression analysis revealed that WNT5A was expressed in human RMS samples and its expression is more prominent in ERMS [63]. miRNA can also regulate RMS progression that might serve as clinical biomarkers. They can act by directly regulating myogenic-regulatory factors, thereby affecting muscle differentiation [64]. Some miRNAs exert effect on Wnt/β-catenin pathway molecules such as miR-1, miR-133a/b, and miR-206, muscle-specific miRNAs were downregulated in RMS, while the miR-29 family also plays a tumor-suppressive role in muscle differentiation and tumor progression [65,66].

Targeting antagonists is a promising therapeutic intervention against many RMS. Several families of Wnt antagonists have been identified so far, but the oncogenic activity of Dickkopf (DKK) family has been widely researched. DKK, which are soluble proteins, prevent the formation of the Fz–LRP6 complex, by interacting with the LRP 5/6 receptor. When DKK was inhibited, β-catenin was reactivated and focal adhesion kinase (FAK) was consequently modulated, which had a favorable impact on in vitro expression of myogenic markers and decreased the proliferation and invasion of cells. Thus, adoption of treatment modalities based on the reactivation of Wnt/β-catenin signaling, intertwined regulatory pathways shall be taken into consideration in RMS patients [67].

## 7. Wnt Signaling in Wilms Tumor (Nephroblastoma)

Wilms tumor, also known as nephroblastoma, is the fourth most common malignancy among pediatric cancers. It arises in the abdomen and affects children from the 3 to 5 years of age [68,69]. Over the last five decades, the choice of treatments in nephroblastoma patients have been nephrectomy in combination with stem cell transplantation, chemotherapy, and radiotherapy [70]. With the advent of newer therapies, patient outcomes have improved, with more than 90% of patients having a long-term survival rate [71]. However, recurrence and metastasis in some patients lead to unfavorable outcomes [72]. Therefore, identifying specific nephroblastoma biomarkers may aid in the study of the pathophysiology and metastasis process as well as augment the current treatment of nephroblastoma patients.

The Wnt signaling pathway is an important embryological signaling pathway that plays a pivotal role in the development of kidneys [73]. Therefore, it may be important to study the effect of abnormal Wnt/β-catenin signaling on the occurrence of nephroblastoma. Progenitor cells expressing the Wilms tumor suppressor gene, WT1, are induced to differentiate during embryogenesis in response to WNT signaling from the ureteric bud. The WT1 gene is responsible for epigenetic silencing of β-catenin [74]. In hereditary Wilms tumors, clonal loss of WT1 impedes the β-catenin expression and leads to dysplastic nephrogenic rests. CTNNB1 clonal mutations in Wilms tumor are diverted by WT1 mutations. A high selection pressure is seen for CTNNB1 mutations in cells that have lost functional WT1 [75].

DEP domain containing 1 (DEPDC1) is an important modulator of the Wnt pathway, reported as a tumor-related gene that promotes carcinogenesis and is found to be upregulated in numerous cancer types [76]. DEPDC1 facilitates the malignant transformation of nephroblastoma by employing the Wnt/β-catenin signaling pathway, which may be modulated by forkhead box transcription factor 3a (FOXO3a) [77]. Su et al. revealed that DEPDC1 was overexpressed in nephroblastoma and associated with poor prognosis. In addition, other studies have shown that DEPDC1 aids cancer development and growth in lung adenocarcinoma [78], gastric cancer [79], oral squamous cell carcinoma [80], colorectal cancer [81], liver cancer [79] and breast cancer [82]. Another study reported that the activation of the Wnt/β-catenin signaling pathway by DEPDC1 promotes the proliferation and metastasis of cancer [81,83]. In addition to this, overexpression of FOXO3a has been reported to downregulate the expression levels of p GSK 3β, β-catenin, and Wnt3a protein, which are important components of the Wnt/β-catenin signaling pathway, by disrupting the DEPDC1-dependent Wnt/β-catenin pathway [84]. Therefore, DEPDC1 might be explored for its contribution in Wnt/β-catenin modulation and trialed for treatment of nephroblastoma in future. It has been established that miRNA 483 influences Wnt/β-catenin, TGF-β, and TP53 signaling in Wilms tumor. Using gene expression data, five Wilms tumor subgroups were identified in which two of them exhibited definite evidence of the activation of the Wnt pathway, as measured by the presence of β-catenin in the nucleus. They also suggested that these subgroups exhibited a recurrent germline or somatic mutations in genes involved in microRNA biogenesis, such as DROSHA and DICER [85]. There is enough evidence indicating that the abnormal expression of Wnt/β-catenin component induces the development and progression of various cancer malignancies [86,87]. Research inquisitiveness may drive the intent to identify agonists and antagonists of the Wnt pathway for furthering the treatment of nephroblastoma, which could ultimately lead to better long-term outcomes.

## 8. Challenges of Wnt/β-Catenin Targeted Therapies in Pediatric Tumors

**Figure 4:**
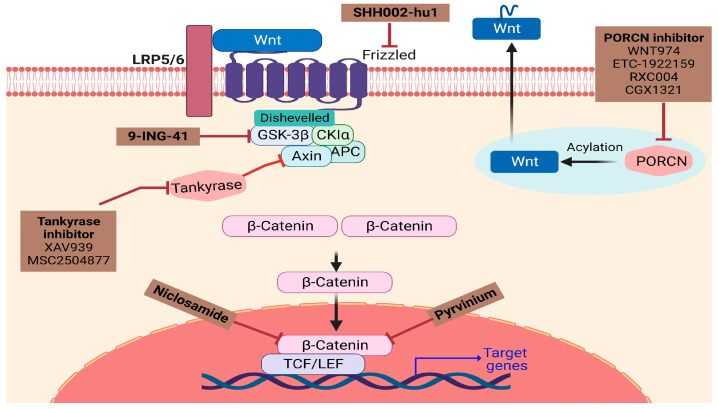
An illustration of the specific Wnt signaling pathway associated inhibitors that have been studied in preclinical and clinical trials for pediatric tumors. There are various inhibitors such as Porcupine (PORCN) (ETC-1922159, WNT974, RXC004, CGX1321) a membrane-bound O-acyl transferase required for palmitoylation of Wnt proteins; SHH002-hu1, a humanized antibody targeting FZD7 receptors; 9-ING-41, a small molecular inhibitor of GSK-3β; Tankyrase inhibitor (MSC2504877, XAV939); Niclosamide, FDA-approved salicyclamide derivative that targets the Wnt/β-catenin pathway; Pyrvinium, a quinoline-derived cyanine dye that inhibits Wnt signaling pathway.

Current research on drug development for Wnt targeted therapies in cancer has been successful, yet there are few FDA-approved drugs in oncology practice [88]. Dose optimization, monitoring the long-term effects, increasing life span, and ensuring a good quality of life must remain the prime concern in pediatric patients before proposing novel drug therapy. The preclinical studies and clinical trials of various inhibitors targeting the Wnt pathway in pediatric tumors are still in process (Figure 4). Many interconnecting components of this pathway will allow for several potential targets for therapy.

There are various upstream modulators of the Wnt/β-catenin pathway which act on components of the pathway near the cell membrane, such as porcupine inhibitors, axin/tankyrase inhibitors, axin inducers, FZD blockers, R-spondin/LGR5 inhibitors, and DKK1 inhibitors. Similarly, downstream modulators such as β-catenin inhibitors and GSK-3 inhibitors act directly on β-catenin or components that act on β-catenin. Antibody-based or decoy-receptor drugs targeting ligands or receptors have been developed as β-catenin inhibitors for preclinical studies and/or clinical trials [89]. β-catenin interacts with TCF/LEF transcription factors to regulate the expression of Wnt target genes within the nucleus. Disrupting this interaction serves as a potent strategy to block this pathway. In this regard, molecules such ETC-1922159 in combination with Pembrolizumab (NCT02521844) have entered clinical trials for the treatment of adult solid tumors. Pembrolizumab is an immune check point inhibitor and is well endured in pediatric patients with impressive anti-tumor effects in relapsed or chemo-resistant Hodgkin lymphoma, which are concordant with results in adult patients [90]. Sunitinib [91], GANT-61 [92], Niclosamide [32], and Sorafenib [93] have been identified for their role in treatment of various pediatric tumors, as potential molecules that act by inhibiting Wnt/β-catenin signaling pathway. Other such potential drugs targeting the Wnt pathway and its components are listed in Table 1.

Taking into consideration the pan system and intricate involvement of Wnt/β-catenin signaling in maintaining normal tissue homeostasis, it is irrefutable that targeting this pathway will yield unwanted byproducts [112]. Although targeting nuclear β-catenin in aberrant canonical Wnt signaling in cancers is a promising approach, the main challenge is to overcome limitations in its druggability [113]. An in-depth analysis of previously available molecular targets may expedite the development of targeted therapies against Wnt/β-catenin signaling in cancers and may possibly overcome the limitations in preexisting molecules [1]. Employing novel strategies such as immunotherapy, synergistic pharmaceutical compounds, lower-weight mAbs, and other similar approaches will provide us with fundamental advancements that will enable us to make precision medicine targeting the canonical Wnt signaling [114].

## 9. Conclusions

The Wnt/β-catenin signaling pathway has generated substantial interest in the field of cancer research because of their extensive involvement and intensive roles in regulating numerous aspects of cancers, including the initiation, development, progression, and diagnosis [115,116]. The focus of this review was to identify specific aberrations affecting the Wnt pathway in different pediatric cancer and understand their therapeutic, diagnostic, or prognostic potential. This review helps to identify specific compounds that may be used in the development of medications and other substances with targeted effects on that biological process. Although there are many Wnt components that can be targeted in development and homeostasis that create challenges for drug specificity and safety in pediatric population [4]. Significant work is still needed to be performed for the identification of distinctive aberrations and strategies for bed to bedside translation. This review hence further corroborates that the Wnt network is both a crucial mediator of proliferation and self-renewal of cancer cells, thus emerging as an enticing target for new drugs [117]. Using multivalent combination therapy to inhibit different pathways provides promising research insights for creating advanced treatments [118].

To surmise, this review intended to systematically exhibit the role of the Wnt/β-catenin signaling pathway in pediatric cancers and was aimed at generating a thorough awareness of current challenges and crises. Additionally, the goal of this review was also sought to provide an idea of future research objectives about the issues and insights that should be considered when developing better-targeted therapies against the Wnt/β-catenin signaling pathway in pediatric tumors.

## Figures and Tables

**Table 1:** List of pharmacological agents of Wnt/β-catenin signaling pathway in pediatric tumors.

	Drug	Mechanism of Action	Disease	Literature
1	ETC-1922159 + Pembrolizumab	PORCN inhibitor, inhibits the extracellular secretion of Wnt	Solid Tumors	[90]
2	ONC201	Reduces the expression of several Wnt pathway components	Solid tumors Neuroblastoma	[94,95,96]
3	9-ING-41	Maleimide-based ATP-competitive small molecule GSK-3 inhibitor	Pediatric cancer, Neuroblastoma	[88,97]
4	Rapamycin	Suppresses the mTOR and Wnt/β-catenin pathways, induces autophagy	Neuroblastoma	[98,99,100]
5	MSC2504877	Tankyrase inhibitor	Neuroblastoma	[101]
6	IWP Compounds	PORCN inhibitor	Neuroblastoma	[102]
7	XAV939	Tankyrase 1(TNKS1) inhibitor	Neuroblastoma, Retinoblastoma	[103,104]
8	Sunitinib	Inhibits TGF-β1-induced Wnt signaling	Neuroblastoma, Retinoblastoma	[1,105]
9	GANT-61	Inhibits Wnt/β-catenin and Notch signaling pathways	Neuroblastoma Rhabdomyosarcoma	[92,106]
10	Niclosamide	Inhibits Wnt/β-catenin signaling	Retinoblastoma	[32]
11	* CircTET1	Inhibits WNT/β-catenin signaling	Retinoblastoma	[38]
12	Cyclopamine	Inhibits β-catenin	Embryonal Rhabdomyosarcoma	[107,108]
13	Sorafenib	Inhibits WNT/β-catenin signaling	Rhabdomyosarcoma, Wilms Tumor	[93,109]
14	Pyrvinium	Inhibition of β-catenin gene transcription	Wilms tumor	[110]
15	SHH002-hu1	FZD7 inhibitor	Wilms Tumor	[111]

* Circ: Circular, PORCN: Porcupine; IWP: Inhibitors of Wnt production; GSK-3: Glycogen synthase kinase-3; TGF-β1: transforming growth factor beta 1; FZD: Frizzled 7.
